# Beyond equipoise: why automated ventilation trials should measure what really matters

**DOI:** 10.62675/2965-2774.20260475

**Published:** 2026-05-18

**Authors:** Marcus J Schultz, Laura Buiteman-Kruizinga, Ary Serpa, Thomas Heidegger, Frederique Paulus

**Affiliations:** 1 Medical University of Vienna Division of Cardiothoracic and Vascular Anesthesia & Critical Care Medicine Department of Anesthesia, General Intensive Care and Pain Management Vienna Austria Department of Anesthesia, General Intensive Care and Pain Management, Division of Cardiothoracic and Vascular Anesthesia & Critical Care Medicine, Medical University of Vienna - Vienna, Austria.; 2 Reinier de Graaf Hospital Department of Intensive Care Delft Netherlands Department of Intensive Care, Reinier de Graaf Hospital - Delft, Netherlands.; 3 Hospital Israelita Albert Einstein Department of Critical Care Medicine Sao Paulo SP Brazil Department of Critical Care Medicine, Hospital Israelita Albert Einstein, Sao Paulo (SP), Brazil.; 4 HOCH Health Ostschweiz Department of Anesthesia, Intensive Care and Emergency Medicine Switzerland Department of Anesthesia, Intensive Care and Emergency Medicine, Spital Grabs, Grabs, HOCH Health Ostschweiz - Switzerland.; 5 University of Applied Research Faculty of Health Amsterdam Netherlands Faculty of Health, University of Applied Research - Amsterdam, Netherlands.

Two major trials of automated ventilation published in 2025 tell a familiar story. PROMIZING, published in the New England Journal of Medicine, found that proportional assist ventilation (PAV+) did not differ from pressure support ventilation in time to successful liberation.^(1)^ ACTiVE, published in JAMA, showed that INTELLiVENT-ASV produced no difference in ventilator-free days at 28 days compared with protocolized conventional ventilation.^(2)^ Both trials were methodologically rigorous, adequately powered, and measured the endpoints we have used for decades: ventilator-free days, duration of ventilation, and mortality. These endpoints remain essential and should not be displaced.

But placed in a broader context, these findings fit a well-established pattern. As in many previous mechanical ventilation trials, PROMIZING and ACTiVE showed no improvement in these established patient–centered outcomes. After three decades of ventilation research, we have repeatedly shown that different ventilation strategies often produce equivalent patient outcomes. While landmark trials like ARMA^(3)^ and PROSEVA^(4)^ established a clear benefit of lung-protective ventilation and prone positioning, most subsequent studies comparing tidal volumes, positive end-expiratory pressure (PEEP) strategies, and recruitment maneuvers have yielded neutral results. In this respect, PROMIZING and ACTiVE are not exceptions but confirmations of a broader trend.

However, these two trials also evaluated technologies that were developed with an additional promise in mind: to reduce the cognitive and manual burden of mechanical ventilation through continuous, automated adjustment of ventilator settings. In other words, beyond potential effects on patient-centered outcomes, these systems were designed to improve the efficiency and sustainability of care delivery. Yet neither trial quantified caregiver workload, nursing time requirements, or system-level efficiency. An alternative interpretation, therefore, is not that automation lacks value, but that it was evaluated using outcomes not designed to capture these operational effects.

For years, this distinction might have seemed academic. But the COVID-19 pandemic exposed a truth that the critical care community had long ignored: we cannot meet the requirements of modern critical care with the healthcare workforce we have. Intensive care units (ICUs) failed not because ventilators were scarce, but because there were not enough people to operate them. Since the pandemic, nurses have left in unprecedented numbers, and ICUs have closed beds even though equipment is available.^(5,6)^ The crisis revealed and widened what workforce analyses had projected years earlier.

In this context, PROMIZING and ACTiVE represent missed opportunities. Both tested technologies are explicitly designed to reduce the cognitive and manual burden of mechanical ventilation. Automated closed-loop systems continuously adjust ventilator settings based on real-time physiologic feedback, theoretically allowing clinicians to manage more patients safely. Whether this design goal translates into lower caregiver workload, reduced cognitive burden, or different staffing requirements in practice remains unknown, because these outcomes were not measured in these trials. Ventilation strategies that yield similar clinical outcomes can impose very different demands on caregivers, and these operational differences increasingly determine what is feasible in real-world ICU practice.

While fixated on ventilator-free days and mortality, we have ignored an outcome that increasingly determines whether patients receive care at all: caregiver burden. Every ventilation strategy exists within an ecosystem of nursing, respiratory therapy, and medical supervision. Some approaches demand near-constant bedside presence, frequent adjustments, and complex monitoring; others can run safely with minimal intervention. Yet our studies treat these as equivalent if mortality rates match. Importantly, automation could plausibly decrease, increase, or simply redistribute workload, given the need for training, monitoring, and validation of automated systems. Without systematic measurement, it is impossible to know which of these effects predominates in real-world practice.

This blind spot is becoming untenable. In many ICUs, and in particular in resource-limited settings, the limiting factor is no longer ventilator capacity or medical knowledge but human availability. This does not imply that technology can substitute for adequate staffing or expertise. Rather, when human availability is the binding constraint, even modest changes in per-patient workload may influence what is feasible at the margin. Whether automated systems meaningfully shift this balance, in either direction, is an empirical question that remains unanswered. When two ventilation strategies yield identical mortality but one requires twice the nursing time, they are not equivalent from a system perspective. Small efficiency differences determine whether hospitals keep beds open or turn patients away.

Consider what automation promises (Figure 1). Proportional assist ventilation and INTELLiVENT-ASV were developed precisely to reduce the need for constant clinician intervention. They automatically titrate PEEP, fraction of inspired oxygen (FiO_2_), pressure support, and respiratory rate based on continuous monitoring of peripheral oxygen saturation (SpO_2_), end-tidal carbon dioxide (CO_2_), and respiratory mechanics. In theory, this should free clinicians to manage more patients or perform other critical tasks. Whether this theoretical advantage translates into lower workload or cognitive burden in practice remains uncertain, and this uncertainty reflects an evidence gap rather than a settled conclusion. Yet we have no data on whether this theoretical advantage translates into measurable reductions in workload, cognitive burden, or staff requirements.

**Figure 1:**
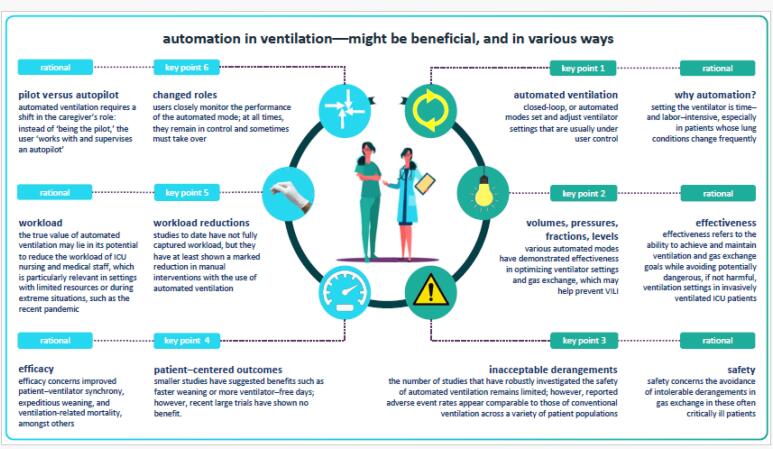
Conceptual overview of automated ventilation in critically ill patients.

Automated ventilation (e.g., PAV+ or INTELLiVENT-ASV) achieves patient-centered outcomes comparable to conventional ventilation, while theoretically reducing the need for continuous manual adjustments and cognitive effort at the bedside. These systems are designed to support workforce efficiency and operational sustainability, particularly in settings where clinician availability is limited. The magnitude and direction of these potential benefits remain uncertain, as they have not been systematically measured in clinical trials.

The ACTiVE trial did show that automated ventilation improved ‘ventilation quality’: patients spent more time within target ranges for tidal volume, pressure, SpO_2_, and end-tidal CO_2_.^(2)^ This finding hints at what we are not measuring: if automated systems maintain patients within safe parameters with less manual adjustment, they may allow safer care with fewer resources. However, any system-level effects could be transient or offset by new sources of complexity, and quality of care must remain central. We therefore do not argue that automated ventilation delivers durable efficiency gains, but rather that, if they exist, such effects should be rigorously evaluated alongside patient-centered outcomes. But without quantifying the caregiver time saved, we cannot evaluate whether automation's real value lies not in improved patient outcomes but in improved system sustainability.

These realities force uncomfortable questions. Should we accept clinically equivalent treatment if it allows us to care for more patients overall? When resources are finite, is a therapy that requires half the nursing time more valuable than one with marginally better individual outcomes? Traditional medical ethics, focused on the clinician–patient relationship, offers little guidance. Public health ethics recognizes that maximizing access may be more ethical than optimizing individual treatment when resources are constrained.^(7)^

Several barriers hinder this shift. Regulatory frameworks emphasize patient safety over system efficiency. Academic incentives reward reductions in mortality, not in workload. Measuring caregiver burden requires complex time-and-motion studies. Medicine's culture resists acknowledging that resource constraints should influence treatment decisions, even when they already do invisibly.

We propose fundamental reorientation of critical care research. The next trials of automated ventilation should include caregiver burden as a primary or co-primary endpoint. This could be operationalized as ‘caregiver-viable days’: the number of days when staff and systems can safely deliver care, or through validated measures of nursing workload or cognitive load scores. We recognize that workload is multidimensional and difficult to disentangle, encompassing time, cognitive load, coordination demands, and supervisory requirements. Approaches such as time-and-motion studies, validated workload or cognitive load instruments, and staffing metrics should therefore be viewed as complementary tools that themselves require further validation and methodological development.

Overcoming these barriers requires both cultural and structural change. Regulators should permit evaluation of system-level benefits alongside patient outcomes. We need validated instruments to quantify caregiver workload, cognitive load, and burnout risk. Economic analyses should routinely include staffing costs and training requirements. Reimbursement structures should recognize that technologies reducing caregiver burden provide system-level value even without improving individual outcomes. Medical education should acknowledge that sustainable care for populations sometimes requires accepting constraints on individual optimization.

Reorienting research toward sustainability does not mean abandoning patient welfare. It means recognizing the two are inseparable. Burned-out, overstretched caregivers cannot deliver optimal care. The next advance in respiratory care may not be a new mode or setting, but the recognition that simplicity and sustainability are lifesaving too.

PROMIZING and ACTiVE taught us that automated ventilation systems achieve outcomes equivalent to conventional management. Now we must ask: are they operationally superior? We have learned which treatments work equally well. Now we must determine which ones our workforce can sustain, because the most effective treatment in the world is worthless if there is no one left to deliver it.

## Data Availability

The contents are already available.
